# Social Scientific Analysis of Human-Animal Sexual Interactions

**DOI:** 10.3390/ani10101780

**Published:** 2020-10-01

**Authors:** José María Valcuende del Río, Rafael Cáceres-Feria

**Affiliations:** Departamento de Antropología Social, Psicología Básica y Salud Pública, Universidad Pablo de Olavide de Sevilla, Carretera de Utrera Km 1, 41013 Sevilla, Spain; jmvalrio@upo.es

**Keywords:** bestiality, human-animal sexual relations, zoophilia

## Abstract

**Simple Summary:**

Sexual relations between humans and animals have been fundamentally approached as a pathology within the fields of health science and biomedical science. Such research has not taken into account the contextual and symbolic nature of so-called zoophilia. Very few studies have analysed zoophilia from the perspective of the social sciences. The taboo surrounding these practices has silenced a reality that is present in countless societies. This paper examines the different ways in which this phenomenon has been tackled in disciplines such as anthropology, sociology and history, allowing us to understand the different meanings and significances of zoophilia depending on the historical and cultural context. The category of zoophilia encompasses a plural reality. Factors such as age, gender or the unequal significance of animals help us to understand a complex phenomenon, which calls into question the radical separation between humans and animals, as highlighted by more recent research within the field of anthropology.

**Abstract:**

An ontological shift has led to a revitalisation of the research area that, within the social sciences, deals with the interactions between humans and animals. However, there are topics which are still taboo: interspecies sexuality. Sexual practices between humans and animals have been fundamentally analysed from a medical perspective, failing to consider the influence of cultural context. Departing from a thorough bibliographical revision, here we revise the approaches that, both from sociology and anthropology, have been used to analyse this phenomenon from different perspectives, including bestiality, zoophilia, and zoosexuality.

## 1. Introduction

In the post-modern age, animals are no longer viewed as mere objects, subject to human designs, and have acquired much greater prominence in their own right. This process is clear at a social level, in the rise of animal rights movements, and in growing legal, cultural, and ethical debates [1]: How far should animal rights extend? To what extent can these rights be comparable to human rights? Further, ultimately, how does this recognition affect the very consideration of what it is to be human?

The development of certain species cannot be understood without human action [2]. Similarly, human experience cannot be understood without analysing our economic, symbolic, political, and even purely physical interactions with animals. Social sciences in general, and anthropology in particular, have played an important role in recent years in the theoretical questioning of boundaries between human and non-human [3,4,5,6,7]. Within the field of the social sciences, there is growing concern to understand ‘the social’ dovetailed with ‘the environmental’; this implies rethinking relations between humans and the other animate and inanimate beings with which we interact [8]. This questioning ultimately entails rethinking the way in which we investigate. This question is raised in Kohn’s emblematic work [9], which signals the need to “shift ethnographic practice towards a process that would situate worlds, which were previously too human, within a broad and emerging series of interrelations that would exceed the purely human” [10].

This move to question the centrality of ‘the human’ has two planes. The first refutes the boundaries between species, even questioning the very validity of the term species [11]. The second queries the frontiers between machine and human [12]. Thus, we are entering what several authors have begun to define as the post-humanist age [13,14,15,16]; this generates not only scientific debates but also moral and ethical reassessments [17].

That said, in this resignification of human-animal relations within the social sciences, there are, nonetheless, certain aspects which have been, and indeed continue to be, pushed to the background, from which the old and the new perspectives, equally, shy away. This is the case with sexual practices between humans and animals.

In this study, we are interested precisely in examining the different approaches generated within the social sciences in relation to human interspecies sexuality.

We are aware that a subject such as this attracts a certain degree of social rejection, which encompasses all manner of moral debates. It is not our aim here to examine such discussions in depth, but instead to analyse how subjects and constructed practices have been placed beyond the borders of normal sexuality, despite ethnographic evidence which shows the unequal signification of such practices depending on the social and historical context.

## 2. Theoretical Approaches to Sexual Relations between Humans and Non-humans

Sexual practices with animals have not been a key focus of attention within the social sciences in general, and anthropology in particular. Perspectives on sexuality have been profoundly influenced by biological, medical, and psychological discourses, but also by the moral prejudices and religious beliefs of the researchers themselves.

Sexuality has been interpreted in terms of reproduction, without taking into account other meanings and significances [18]. If this concealment has been evident in non-human interspecies relations, the silence surrounding human involvement in such practices is hardly surprising. Human-animal sexual practices not only call into question the model of heteronormativity, but also overstep the boundary of what is considered strictly human [19]. Shedding light on these ‘sexual relations’ ‘de-sacralises’ human sexuality. It harks back to an animality denied in anthropocentric visions, which represent humans as qualitatively different from other animals. It is no coincidence that these types of practices are recognised firstly in those considered ‘less’ human. Evidence of human-animal sexual relations has been used to stake out the boundary between ‘barbarians’ and ‘civilised’ peoples. Chroniclers who recounted processes of ‘colonisation’ regularly described all the practices that legitimised domination of the animalised ‘other’, a being that must be taught, dominated, and colonised [20,21,22].

Although awareness of bestiality reinforced the image of the non-Western savage, from the 19th century onwards, it was also used to mark out internal ‘primitives’, members of the population who did not meet the standards of urban life: peasants. In Kinsey’s emblematic work [18] on sexuality, zoophilia in America was firmly situated in the rural world. For this author, the rural context helped to explain zoophilia, since it is an environment with strong sexual control and little access to women. At no time was it suggested that it might be a voluntary option or a preference: contact with animals was considered as a replacement of sexual relations with women [18]. This same interpretation can be found in research about bestiality in Sweden during the modern age [23,24]. As noted by Miletski [25], there is a widespread stereotype about zoophilia as the practice of poor and ignorant peasant men. However, what happens in the case of civilised urban societies?

The literature about bestiality and zoophilia is much scarcer in urban contexts, prior to the arrival of the Internet, at any rate, as we shall see later on. Urban zoophilia is far less documented, and the theoretical approach taken is also different. Practices with animals in contexts of colonisation and in rural areas are understood as a moral question. They take on a collective character that transcends the individual, thus justifying ‘educational’ and ‘moralising’ actions. In the urban world, zoophilia is no longer a sociological and anthropological problem, and instead is treated as an individual psychiatric disorder. What is understood as immorality among ‘primitives’ is seen as mental disturbance among ‘modern’ populations.

The evolution in the terms used to denote human-animal sexual practices shows a change in meaning that is intrinsically linked to transformations in social models of sexuality; in other words, the social systems of desire management. On the basis of these models, normality and deviance are constructed, and certain practices are rewarded or penalised, as suggested by Rubin [26], depending on their proximity or distance from the ‘ought to be’ norm of sexuality.

If we look at the ethnographic data, we see that these practices are not confined to a specific society, but rather are spread all over the planet and can be found throughout history, from pre-historic times to the present day [27,28]. However, the reasons for rejecting sexuality with animals are not universal and have not remained constant over time.

In some cases, such relations have been condemned by law, and also by religion. Hence, Leviticus (20:15) states: “if a man lieth with a beast, he shall surely be put to death: and ye shall slay the beast.” [29]. In Europe, until the late 19th century, bestiality was akin to sodomy. These terms had a clear moral component and were penalised because they were considered a sin and, therefore, in that context, a crime. However, from the 19th century onwards, with the development of psychiatry, there was an important shift in the way these kinds of relations were treated. ‘Perversion’ and ‘immorality’ were transferred from the practice to the person. Behaviours were essentialised and linked to ‘sick’ bodies. As signalled by Foucault [30], throughout the 19th century, medicine offered the bourgeoisie new ways of legitimising social control over dissidents in general and over sexual dissidents in particular. This process occurred not only with regard to sexuality with animals but also in other non-reproductive sexualities (same sex relations, masturbation, fetishism.) [26,30,31].

Medicine and psychiatry brought to light countless peripheral sexualities that were stigmatised as illnesses [26,30]. In 1886, the German psychiatrist Krafft-Ebing drew a distinction between bestiality and zooerasty or zoophilia. He used the term bestiality for practices aimed exclusively at satisfying sexual desire through the use of other species. Bestiality is explained either by psychopathological conditions or by ‘moral baseness’: excessive sexual desire or the lack of opportunities to satisfy this desire ‘naturally’. The terms *zooerasty* or *zoophilia,* on the other hand, refer to pathological behaviours that imply sexual and emotional attraction to animals [32].

However, there is another group of cases falling well within the category of bestiality, in which decidedly a pathological basis exists, indicated by heavy taint, constitutional neuroses, impotence for the normal act, impulsive manner of performing the unnatural act.

Following this distinction, Ellis [33] stated that bestiality would imply that “the individual is fairly normal, but belongs to a low grade of culture”. Zoophilia, on the other hand, would apply to “the other in which he may belong to a more refined social class, but is affected by a deep degree of degeneration”.

The term zoophilia gradually gained ground over the label of bestiality. Zoophilia was included in the Diagnostic and Statistical Manual of Mental Disorders (DSM) compiled by the American Psychiatric Association (APA) [34] as a paraphilia. Zoophilia appears for the first time as a paraphilia in the 3rd edition of the Diagnostic and Statistical Manual of Mental Disorders (DSM-III) in 1980. In the revised 3rd edition of the DSM-III-R (APA, Washington, D.C., USA, 1987) it is classified as Paraphilia NOS or Paraphilia (Not Otherwise Specified) [35].

The pathologisation of zoophilia implies that any treatment of this issue has been marked fundamentally by a medical/psychiatric orientation [36,37,38,39,40,41]. As this discourse links zoophilia to pathology, the literature developed has analysed the connections between these practices and a series of aggressive and violent behaviours within the field of forensic and criminological studies [42,43,44,45,46].

Far less research has examined zoophilia from the perspective of the social sciences. One important study in terms of its repercussions was that of Midas Dekkers [27], which analyses sexual relations with animals in psychology, law, literature, art, and advertising. The frequent artistic depictions of sexual practices between humans and non-human animals at different points in history and across different cultures have sparked particular interest: one well-known example is the sculptures in the Hindu-Jain temples of Khajuraho in India (10th–11th centuries), which, together with a variety of sexual behaviours, represent copulation between people and animals [47,48]. Some historical studies have also tackled bestiality, particularly through the analysis of legal proceedings against those accused of such practices [23,24,49]. Educational historical studies have highlighted the widespread incidence of these practices in different contexts and historical periods. However, these are still largely a mere anecdotal compilation of examples, which do little to further our understanding of their social significance and meaning [50].

Fragmented and decontextualised data, methodological difficulties in accessing informants that are not marked by exclusion (as is the case in criminological studies), together with the moral prejudices of the researchers themselves, and the prevalence of the medicalised vision are the main reasons behind the lack of interest shown by sociology and anthropology in this issue. However, this has begun to change in recent years on account of the increasing visibility of these sexual practices on the Internet.

## 3. Contributions of Anthropology and Sociology in Studies about Zoophilia

Anthropological research on sexual relations between animals and humans is scarce [51]. Ethnographic studies merely contain anecdotal references [52,53,54,55,56,57]. The starting premise in such approaches is that zoophilia is a deviation from normative sexuality, a fact that is questioned in other works that approach this phenomenon from a perspective that is not necessarily pathologising.

In his groundbreaking work on the anthropology of sexuality, *The Sexual Life of Savages in the North West of Melanesia* [52], Malinowski devotes barely one page to such practices. He includes them within the category of contemptible sexualities (along with exhibitionism, homosexuality, masturbation, anal and oral sex), substitutes for the adequate exercising of sexual impulse. He includes just one case of sexual relations between a man and a dog, a behaviour that is seen as ridiculous and classed as disgusting and unsatisfactory [52]. He also notes that in the past, this behaviour was punished more severely. The person involved was accused of witchcraft and the animal was sacrificed. He is quick to compare bestiality with ‘inversion’, affirming that the Trobiand people consider it even more absurd.

Even Evans-Pritchard does not tackle this issue, in spite of his interest in sexuality and his studies of societies such as the Zande and the Nuer, in which relations between animals and humans are very close. His work contains just one footnote about a case involving a cow. The old man involved, overcome by shame and regret, sacrificed the animal and also cut his own finger off with a spear as a way of atoning [56].

Among the research that has dealt with zoophilia more precisely is the study conducted by Devereux [58] on the Mohaves of North America, and LeVine’s [59] work on the Kisii people of Kenya. More recently, Marie-Christine Anest [60] has investigated zoophilia in Cyprus and Crete. As noted by Devereux [58], relationships with animals are not the same in all cultures, and they are not identical with all species. This author explains that, for the Mohaves of North America, men and animals were not originally differentiated [58]. Ellis [33] holds the same opinion, considering that these kinds of practices are favoured among ‘primitive’ peoples on account of their conception of nature, in which there are no great barriers between humans and animals. The same would hold true among peasants on account of their familiarity with their beasts.

Although some authors highlight familiarity and proximity with animals when explaining human-animal sexual practices, others show how ‘closeness’ acts inversely. Ruelland [61], for example, when examining the Tupuri people, notes that zoophilia is akin to incest. Proximity to certain animals on occasions translates into rejection of sexual contact, depending on the significance of the animal, but also on other variables, which are equally fundamental when it comes to understanding how, with which species and in which contexts sexual relations are permitted or not.

Not all species have the same worth or are considered appropriate for sexual relations. For Devereux, the Mohaves and Yuma people only conceive of sexual relations with mares, or female donkeys, cows, or calves. In the cases analysed in Turkey, sex is only permitted with animals that are not eaten, such as dogs and donkeys [62,63]. Furthermore, it is not understood as an appropriate sexuality for all individuals, nor is it admissible under all circumstances. Zoophilia often reproduces heteronormative rules, and relations with animals of the same sex are not accepted, as is the case in the north of Costa Rica [64].

The research conducted in the areas of zoophilia and bestiality notes that variables such as age, gender, and social position influence the acceptability of such sexual practices. References to cases of zoophilia and bestiality most commonly focus on men. This is hardly surprising, since this androcentric vision is widespread in studies about sexuality. However, we know that women in these ‘traditional’ contexts have also engaged in such practices. Female bestiality is usually associated with animals linked to the domestic sphere, such as dogs [32,65,66]; it is no coincidence, therefore, that in many cultures, a man engaging in sexual conduct with female dogs is considered unthinkable or grotesque [58]. In the north of Costa Rica, whereas sexuality with female pigs or donkeys is seen with a degree of normality, sexual relations between teenage boys and female dogs are rejected and ridiculed [64]. In Greece, dogs are prohibited for such practices, considered impure, and there is fear of the diseases that might be contracted [60]. Among the Inuit peoples, sexual relations with dogs were found among men and women alike. Male sexuality with female dogs was regulated: it had to be outdoors, never inside the home, and the animal had to be in heat [63].

As we see through these cases, each social context defines its own rules in relation to the species permitted for sexual relations. In contrast to the vision that presents sexuality in general, and sexuality with animals in particular, as an individual reality, research into this phenomenon shows that sexual practices with animals has a normative and, therefore, collective nature. The different societies in which such practices have been investigated call into question the dominant vision of sexuality with animals as an excessive practice, lacking in any regulation. We should not forget that legal codes are only one means of way of regulating and that custom also defines what should or should not be done within the sphere of sexuality.

Ethnographic data about sexual practices with animals highlight a series of correlations. The first is the normalisation of sexual practices during childhood and adolescence. The second is the connection between sexual practices with animals and masculinity. These practices can be associated on occasion with male rites of passage. In Sweden, in the 17th and 18th centuries, sexual relations with animals were widespread. Liliequist [23] argues that the high number of cases involving minors under the age of 15 was linked with the exploration of sexuality. Rydström [24] also believes that bestiality in Sweden in the late 19th and early 20th centuries was a common behaviour among teenage boys aged between 15 and 17, and that it was sometimes a collective practice. This author suggests that such behaviours can be understood as a form of sexual experimentation in a context in which animal sexuality was more visible, whereas human sexuality was discrete and shameful.

Among the Kisii people of Kenya [59], sexuality with animals among minors under the age of sixteen is viewed with a certain indulgence. It is understood that boys are putting their sexual prowess to the test. However, after this age, the same practices are censured and punished. John Money [67] notes that among the pre-Columbus peoples of the Caribbean coast of Colombia, young men often acquired their sexual skill for marriage through sexual relations with donkeys. This is not simply an historical practice; in the present, on Colombia’s Caribbean coastline, this kind of sexual contact still occurs, to such an extent that the inhabitants of this area are known within Colombia as *comeburras* (literally, “donkey eaters”, meaning they have sex with donkeys) [68]. At a very early age, seven or eight years old, children begin to have their first sexual experiences with female donkeys. They might even pay for sex with these animals [69]. Elsewhere in Central America, these same practices have also been a common occurrence. In 2004, we were able to interview several men in the north of Costa Rica, in a settlement close to the border with Nicaragua, who recounted how young boys frequently initiated their sexuality with animals. It was something spoken about among men; a secret they did not share with women. There, this form of sexuality became concealed from the 1980s onwards, when outsiders who judged these practices as depraved first arrived in their communities.

In Mexico, cases have been documented of pubescent teenagers who engaged in group sexual relations, practices such as masturbation and sex with various animal species—female donkeys, female calves, hens, turkeys, and nanny goats [70]. The same is true in Honduras [71].

Human-animal sexual relations have also been recorded in certain indigenous societies of the Amazon. According to Erikson [72], among the Matis people of the Brazilian Amazon, adult males go out into the rainforest to satisfy their sexuality with sloths, a species they frequently domesticate. This is not an exceptional case since, as this author notes, “Zoophilic practices are no less frequent in the Amazon forests than in the countryside of Europe” [72], indicating certain authors who provide data in this regard: Edeb, among the Aché people of Paraguay [73]; Morey and Metzger among the Guahibo people of the Orinoco Plains [74]. Sexual practices with female dogs are also documented among the Karitiana people of Brazil [75].

The Mediterranean is also no exception, as shown by French anthropologist Marie-Christine Anest [60] in Crete and Cyprus. Even as recently as the 1980s, young boys and teenagers, between the ages of 6 and 17, initiated their sexuality by engaging in sexual conduct with female donkeys, sows, nanny goats and birds. In Greece, as in other parts of the world, these practices were considered a secret between men. They were experiences that were accepted during youth, but which had to be abandoned when the boy reached adulthood. In Anatolia, Turkey, sexual contact between teenage boys and female donkeys was somewhat tolerated in rural communities [76,77]. The Finnish sociologist Edward Westermarck [78] states that in parts of Morocco, some boys before they reach puberty can engage in sexual conduct with donkeys in accordance with the belief that this strengthens sexual potency and will enlarge their penis. However, these same practices among adults were considered ridiculous and rejected. There is practically no research on zoophilia in Spain, with the exception of the text by Coca et al. [79], which documents certain cases in rural populations of western Andalusia. The authors argue that human-animal sexual relations are linked with the learning of sexuality among boys and teenagers. This article highlights certain parallels between the forms of zoophilia noted in studies conducted in the Mediterranean and Latin America. Sexuality with animals can occur, on occasions, during adolescence, almost as a rite of passage, prior to sexual relations between men and women. The research also highlights the ‘secrecy’ of these practices, which are known about within the friendship group, but which are hidden outside of the group, especially from women. Boys interact with animals, they play at ‘being animals’, and they also play at ‘being men’. Sexual practices with other species are interwoven into the construction of masculinity. A negative model that is constructed in contrast to ‘others’: women, lesser men, and boys [80,81]; and also in contrast to animals.

By analysing key anthropological and sociological texts about zoophilia in diverse cultural contexts, we have noted that zoophilia is not always regarded as deviance. There are rules that regulate when, how and where sexual practices with animals are acceptable. However, although these investigations shed some light on certain aspects of interspecies sexuality among humans, they also present certain shadows that need to be addressed. Firstly, when sexuality with animals is discussed, it is done so fundamentally in reference to the sexuality of men. What about the sexual practices of women with animals? This aspect has been rendered practically invisible, just as female sexuality has. However, the lack of research in this regard stands in stark contrast with: (1) the abundance of artistic representations of women engaging in sexual conduct with animals [27] and (2) the growing prominence of women in zoophilic pornography, aimed fundamentally at a male audience. The second question that has barely received any attention is what happens with sexual practices in urban contexts that, now, thanks to the Internet, are being brought into sharp relief as an evident reality. Science is still clearly performative in nature, always finding what it is looking for, in accordance with a social context of knowledge creation that directs our gaze. The voices and silences of science are eminently political. However, in recent years, there has been a reinterpretation of these practices as a new corporality or a new identity is configured: zoosexuality.

## 4. Redefining Relations with Animals

Growing concern surrounding the ways in which humans interact with other species has translated into a global trend towards legal regulation. The rules put in place tend to regulate, from a protectionist perspective, what can be classed as good practices and other practices that should be penalised and even be considered a crime. The ways animals are treated in experiments, the conditions of the enclosures where they are kept, animal husbandry practices, and varieties of animal mistreatment. Broad sectors mobilise in defence of animal rights, demanding a change in their status, which even includes their consideration as legal persons. Aspects previously ignored in different legislations, such as sexual practices, are now being tackled. In fact, at a global level, an increasing number of legislative acts now prohibit any type of sexual relation between humans and non-humans [82,83]. The regulation of these practices gives us a few clues to understanding the change in significance of interspecies sexuality, and ultimately, of animals themselves.

The argument in favour of prohibiting these relations is clear, based on cases of assault and abuse. It is understood that an animal can never give consent, something which would then equate zoophilia with rape [84,85]. Social norms tend to view animals, to all intents and purposes, as social minors that must be protected. This paternalistic vision of animals denies their possibility for agency, from a moral vision that ties in with the biomedical perspective. This is the stance taken by the majority of animal rights defence movements. Such considerations have been largely transferred to criminal law, which now makes provisions for new crimes such as “sexual abuse” of animals [86]. Growing legal regulation raises various issues: (1) to what degree does the consideration of such relations as a crime truly contribute to the protection of non-human animals? and (2) whether certain punishments to condemn certain practices are licit, legitimising other forms of socially accepted abuse, such as those found in the food industry [87]. As this author notes, punitive laws protect certain species whilst ignoring others and the punishment eventually falls onto subaltern social sectors that are criminalised on the basis of ethnocentric considerations.

However, alongside this prohibitionist tendency, we find another trend moving in a very different direction, which argues that sexual relations with animals do not always imply cruelty [11]. Whilst he does not condone zoophilia, Singer considers that, as a taboo, it is irrational, helping to maintain distance between humans and animals. Some authors even argue that zoophilia could be a non-anthropocentric model for relating with other species. This would require a rethinking of interspecies relations, but also a change in the concept of sexuality [88]. This is a minority perspective that argues in favour of the right to maintain affective-sexual relations with animals.

These changes are bringing to light the fact that, obviously, such practices are not confined to ‘primitive’, ‘peasant’ or ‘rural’ societies, nor are they restricted to men or certain ages. Zoophilia is now becoming visible in ‘modern’ societies, ‘urban’ contexts, and also among women. In this regard, the Internet has made a fundamental contribution by connecting people who share certain ways of understanding these practices, structured around a new expression of identity, precisely at a time when sexuality has become one of the core defining elements in individual identity.

Up to the 1960s, as shown [89] in the case of America, the consumption of zoophilic pornography and other variants was through very specialist and selective channels in individualised peep-show booths. However, within the pornographic film industry, the production of zoophilic films now occupies an important place [90]. Widespread access to the Internet has made it easier to consume increasingly repudiated practices. It has disseminated and spread more or less minority practices to the extent of generating a significant sex market that can be adapted to user profiles, practically à la carte [91]. Unsurprisingly, in this context, some countries have developed an emerging industry, which raises new research problems with regard to this part of the pornographic business, in which women take centre stage in the configuration of zoophilic products [90]. Once again, science is concerned with the risks surrounding the possible spread of such ‘perverse’ practices: new forms of objectification of women in zoophilic pornography, access among minors to such practices, and the exploitation of animals turned into mere objects.

However, although the web is a forum for outcry, it has also become a space for confluence and the defence of this sexuality [92,93,94,95]. Individuals who practice this form of sexuality offer positive interpretations, labelling themselves zoosexuals or simply zoos, and creating a ‘‘zoo community’’ [96]. Some members of these networks are becoming aware that their sexual preferences do not constitute an anomaly, merely a different sexuality, and they argue that they are an unacknowledged sexual minority [94,95]. In this context, some researchers are beginning to talk about zoophilia as a sexual orientation [28,97,98]. For Cassidy [98], zoosexuality needs to be understood within the context of recognising new emerging sexual identities that are no longer restricted by a patriarchal system centred on reproduction.

The development of these zoo communities on the Internet has enabled researchers to access such sexual practices more easily [92,93,94,95,99,100]. If we can overcome moral barriers, we have the opportunity to make visible, analyse and understand an approach to human-animal relations that, to date, has been largely ignored. However, zoosexuality is presented as a minority reality, and as a consequence of its illegality in many countries, zoosexuals will become less and less visible. The influence of animal rights defence sectors turns zoophiles and zoosexuals into criminals.

## 5. Conclusions

The social sciences have considered sexuality a peripheral field of research, a fact that becomes particularly evident in the study of zoophilia. Analysis of so-called ‘deviant’ sexualities has been taken on by biomedical sciences, psychiatric sciences, and legal disciplines, in a complementary way. This fact, together with the moral prejudices of the researchers themselves, explains the silences maintained in the social sciences in general and in anthropology in particular. Unsurprisingly, therefore, the first research to tackle such issues peripherally approaches zoophilia as an anecdotal or exceptional behaviour. However, the limited social research conducted so far that has tackled zoophilia has revealed something determined to remain hidden: that these kinds of human-animal relations have been found throughout history and around the world, and that, under certain circumstances and in certain contexts, especially in the case of sexual relations involving men, they have been practised with a certain degree of social acceptance. However, even these investigations remain silent around issues such as zoophilia among women. This absence of research analysing sexual interactions between women and other species stands in contrast with the prominence of women in artistic representations and in the pornography industry, aimed fundamentally at a male audience. Although it seems clear that sexual relations between males and non-human animals are often part of their learning of hegemonic masculinity [79], the androcentric vision of these studies gives us very few clues regarding the significance of feminine zoophilia.

The few studies available, which deal fundamentally with the Mediterranean and Latin America, call into question the univocal nature of human-animal sexual relations and highlight that the same practices can encompass multiple meanings. Zoophilia is not an individual behaviour in the majority of cases investigated, nor does it lack social guidelines, since it has its own rules, defining which animals are or are not appropriate for such practices, the ages at which sexual relations can be had with animals, and which practices are appropriate and inappropriate. It all seems to indicate a clear link between the cultural construction of masculinities and zoophilia. This leads us to question zoophilia as an exception to normative models of sexuality, with which it is often very closely linked, in terms of restrictions to women and in the reproduction of heteronormativity (men must only engage in sexual relations with female animals). This normativity of zoophilia casts doubt on certain commonplaces. Accessibility and proximity to animals might explain why it has been easier to document these practices in the rural world, but this does not explain why certain animals, dogs for example, living in proximity with us are not always considered appropriate for sexual engagement. Overfamiliarity with certain species might indeed contribute to this taboo.

Currently, sexuality with animals is considered especially perverse, not only because it is deemed to degrade the human being, but also because it infringes the rights of animals. Consent becomes the key issue when questioning such practices, although it is true that the development of the online world has increased the visibility of this reality in the urban world as well, allowing groups to connect around a practice that it seen as an element of their identity. Hence, new fields of research and new moral debates are opening up to which the social sciences could make a significant contribution, although to do this we must be able to overcome prejudices that prevent us from tackling a field still colonised, almost exclusively, by medical discourse.
